# Human recombinant erythropoietin (rEpo) has no effect on tumour growth or angiogenesis

**DOI:** 10.1038/sj.bjc.6602846

**Published:** 2005-11-15

**Authors:** M E Hardee, J P Kirkpatrick, S Shan, S A Snyder, Z Vujaskovic, Z N Rabbani, M W Dewhirst, K L Blackwell

**Affiliations:** 1 Department of Pathology, Duke University Medical Center, Box 3893, Durham, NC 27710, USA; 2 Department of Radiation Oncology, Duke University Medical Center, Box 3893, Durham, NC 27710, USA; 3 Department of Hematology–Oncology, Duke University Medical Center, Box 3893, Durham, NC 27710, USA

**Keywords:** erythropoietin, angiogenesis, tumour growth

## Abstract

Tumour hypoxia has been shown to increase mutation rate, angiogenesis, and metastatic potential, and decrease response to conventional therapeutics. Improved tumour oxygenation should translate into increased treatment response. Exogenous recombinant erythropoietin (rEpo) has been recently shown to increase tumour oxygenation in a mammary carcinoma model. The mechanism of this action is not yet understood completely. The presence of Epo and its receptor (EpoR) have been demonstrated on several normal and neoplastic tissues, including blood vessels and various solid tumours. In addition, rEpo has been shown in two recent prospective, randomized clinical trials to negatively impact treatment outcome. In this study, we attempt to characterize the direct effects of rEpo on tumour growth and angiogenesis in two separate rodent carcinomas. The effect of rEpo on R3230 rat mammary adenocarcinomas, CT-26 mouse colon carcinomas, HCT-116 human colon carcinomas, and FaDu human head and neck tumours, all of which express EpoR, was examined. There were no differences in tumour growth or proliferation (measured by Ki-67) between placebo-treated and rEpo-treated tumours. In the mammary window chamber, vascular length density (VLD) measurements in serial images of both placebo-treated and Epo-treated rats revealed no difference in angiogenesis between the Epo-treated tumours and placebo-treated tumours at any time point. These experiments are important because they suggest that the recent clinical detriment seen with the use of Epo is not due to its tumour growth effects or angiogenesis. These studies also suggest that further preclinical studies need to examine rEpo's direct tumour effects in efforts to improve the therapeutic benefits of Epo in solid tumour patients.

Low oxygen tension (pO_2_) promotes the selection of cells with reduced apoptotic potential (Graeber *et al*, 1996), increases the frequency of mutation (Reynolds *et al*, 1996), induces genes promoting angiogenesis (Dachs and Chaplin, 1998) and decreases radiosensitivity (Bedford and Mitchell, 1974). Clinically, tumour hypoxia is associated with poorer outcome. A variety of approaches have been used to reduce tumour hypoxia with the goal of improving the therapeutic response to chemotherapy and/or radiation therapy. A recent promising approach in preclinical models involves the administration of exogenous recombinant erythropoietin (rEpo) (Blackwell *et al*, 2003). Erythropoietin is a glycoprotein growth factor that is essential for erythroid precursors to mature into red blood cells by stimulating growth and preventing apoptosis (Krantz, 1991). It is a clinically important member of the growth factor family, in that it is produced endogenously in the human body and it may be given exogenously in the human recombinant form to treat the anemia associated with several chronic diseases. However, there have been several reports suggesting that both Epo and EpoR can be expressed in various neoplastic tissues, raising the possibility of autocrine pathways. A randomized, placebo-controlled trial of rEpo in nonanemic metastatic breast cancer patients was terminated early because of an unexpectedly higher mortality rate in the group receiving rEpo (Leyland-Jones, 2003). Also, a recent trial involving patients with head and neck cancer found that rEpo did not improve the efficacy of standard therapy (Henke *et al*, 2003). Although the initial results from these two trials are disappointing, their design fails to define a biologic mechanism for why rEpo might lessen treatment effects. This preclinical study examines rEpo's effect on tumour growth and proliferation in two rodent tumour lines and two human tumour lines, as well as its effects on tumour angiogenesis.

## MATERIALS AND METHODS

### Animals

Female BALB/C mice (National Cancer Institute, Washington, DC, USA) were used in the CT-26, HCT-116, and FaDu flank tumour studies. Female Fischer 344 rats (Charles River Laboratories, Raleigh, NC, USA) were used for the R3230 flank tumour studies and the mammary window chamber experiments. Tumour cells were injected into the subcutis of the right quadriceps muscle for flank tumours. Following injection of tumour cells or mammary window surgery, the animals were allowed free access to food and water *ad libitum*. All procedures and experiments were approved by the Duke Institutional Animal Care and Use Committee.

### Cell culture

R3230 (rat mammary adenocarcinoma) and GFP-R3230 (R3230 tumour cells constituitively transfected with green fluorescence protein; as described in (Huang *et al*, 1999)) tumour cells were maintained in DMEM (Sigma, St Louis, MO, USA) medium. CT-26 (murine colon adenocarcinoma) tumour cells were maintained in RPMI (Sigma, St Louis, MO, USA). HCT-116 (human colon carcinoma) tumour cells were maintained in McCoy's 5A medium (Sigma). FaDu (human head and neck carcinoma) were maintained in MEM supplemented with 1% sodium pyruvate and 1% amino-acid solution. All media was supplemented with 10% FBS (Sigma) and 1% penicillin/streptomycin (Sigma).

### rEpo administration and hematocrit measurements

Animals in all studies were administered rEpo (Procrit™, Ortho-Biotech, Raritan, NJ, USA) as a 100 unit ml^−1^ (120 units=1 *μ*g EPO) solution in 0.9% NaCl via an injection into the subcutis of the nape of the neck at a dose of 2000 units kg^−1^ thrice per week. This dosing is similar to a rEpo dose of 60 000–100 000 IU dose^−1^ in humans, as referenced in previous work (Blackwell *et al*, 2003). Hematocrit was determined in all animals by collecting 50 *μ*l of blood in a capillary tube from a tail vein of anesthesized animals, then centrifuging for 1 min and calculating hematocrit from the ratio of the length of packed RBC's to that of plasma.

### Tumour volume

In rats, tumours were measured transversely and the volume calculated using the formula: 
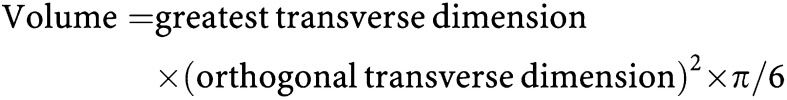
 where all dimensions are in mm. In mice, tumour volume was determined by the formula: 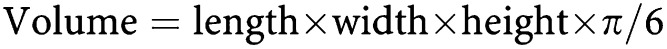 where all linear dimensions are in mm.

### Epo receptor and Ki-67 immunohistochemistry

Standard immunohistochemical techniques were used to stain for both EpoR and Ki-67. Briefly, IHC was performed using 10 *μ*m serial sections of frozen tissue. Sections were fixed in 4°C cold acetone for 10 min. After endogenous peroxidase activity was quenched with 3% hydrogen peroxide for 15 min, tumour sections were blocked with 10% donkey serum for 15 min. Sections were incubated in primary antibodies (anti-EpoR (M-20), 1 : 200 (Santa Cruz Biotechnology, Santa Cruz CA, USA); anti-Ki-67, 1 : 1000 (Novacastra, UK)) overnight at 4°C. The location of the antibody reactions was visualized with 3, 3′-diaminobenzidine tetrahydrochloride (Sigma). For Ki-67 staining, four representative fields at × 40 objective taken from one tumour in each of six animals in both the control and Epo-treatment group were measured. Proliferation index was calculated by manually counting the ratio of Ki-67-positive nuclei to total number of nuclei in each field, and the average proliferation index for each tumour was calculated.

### Mammary window chamber assay

The method used is the same as that described by Shan *et al* (2003). The assay is an orthotopic variation of the dorsal skin fold window chamber, which is the gold standard for serially measuring *in vivo* angiogenesis. The epidermis around either the right third or fourth nipple was removed, leaving an intact nipple and surrounding subcutaneous tissue. The nipple was then removed at its base, exposing the nipple sinus. A 1 mm^3^ piece of Gelfoam (Pharmacia & Upjohn, Kalamazoo, MI, USA) soaked in 3 *μ*l of an R3230-GFP cell suspension of concentration 2 × 10^6^ cells ml^−1^ (total of 6000 tumour cells) was inserted into the nipple sinus. This was then covered with an acrylic window, which was sutured in place.

### Intravital microscopy

The mammary windows were imaged serially using a Carl Zeiss MPS intravital microscope (Carl Zeiss, Hanover, MD, USA) every other day, starting on postoperative day 2 and ending on postoperative day 14. The microscope is equipped with a color video camera (Carl Zeiss ZVS-3C75DE) connected to a PC computer with a frame grabber and Scion Image software (Scion Corporation, Frederick, MD). Fluorescence epi-illumination was provided with a 100 W mercury-arc lamp (AttoArc HBO, Carl Zeiss Inc, USA) and a FITC filter (excitation 450–490 nm and emission 520 nm). Images were obtained at low magnification (objective × 5) and analyzed using Scion Image software. Vascular length density (VLD) was calculated for each image by an observer unaware of the treatment group.

### Statistics

The data on hematocrit, tumour volumes, and VLD were analyzed and treatment groups compared using *t*-test for independent means. In all analyses, two-sided *P*-values less than 0.05 was considered significant.

## RESULTS

### R3230, CT-26, HCT-116, and FaDu tumours express Epo receptor

**Figure 1:**
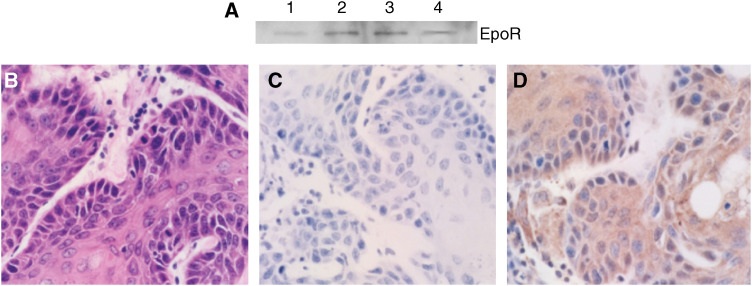
EpoR status of tumour cell lines. Shown in (**A**) is an EpoR Western blot on all tumour cell lines used in this study. Positive staining is seen in all the tumour cell lines. Also shown are H&E (**B**), negative IgG control (**C**), and EpoR (**D**) staining of a representative R3230 flank tumour section taken with a × 40 objective.

To establish relevance for this study, we determined the EpoR status of each of the four tumour cell lines used. All four tumour cell lines used in this experiment expressed Epo receptor by Western blot (Figure 1A). This was confirmed by immunohistochemical staining for EpoR. Interestingly, the strongest staining for the receptor was in perinecrotic regions (Figure 1B).

### Epo increases hematocrit

In the tumour growth studies, baseline hematocrit of mice was 46.5±1.5%. Treatment with Epo produced a hematocrit of 60.9±3.1 *vs* 46.7±2.1% in the control group after 14 days (*P*<0.05). In the mammary window chamber experiments, EPO significantly increased the baseline hematocrit (45.8±2.6) of treated rats by 36% after 8 days and 40% at 14 days compared to placebo-treated rats (*P*=3.2 × 10^−8^ and 4.3 × 10^−9^ at days 8 and 14, respectively).

### Epo does not promote tumour growth or proliferation rate

**Figure 2:**
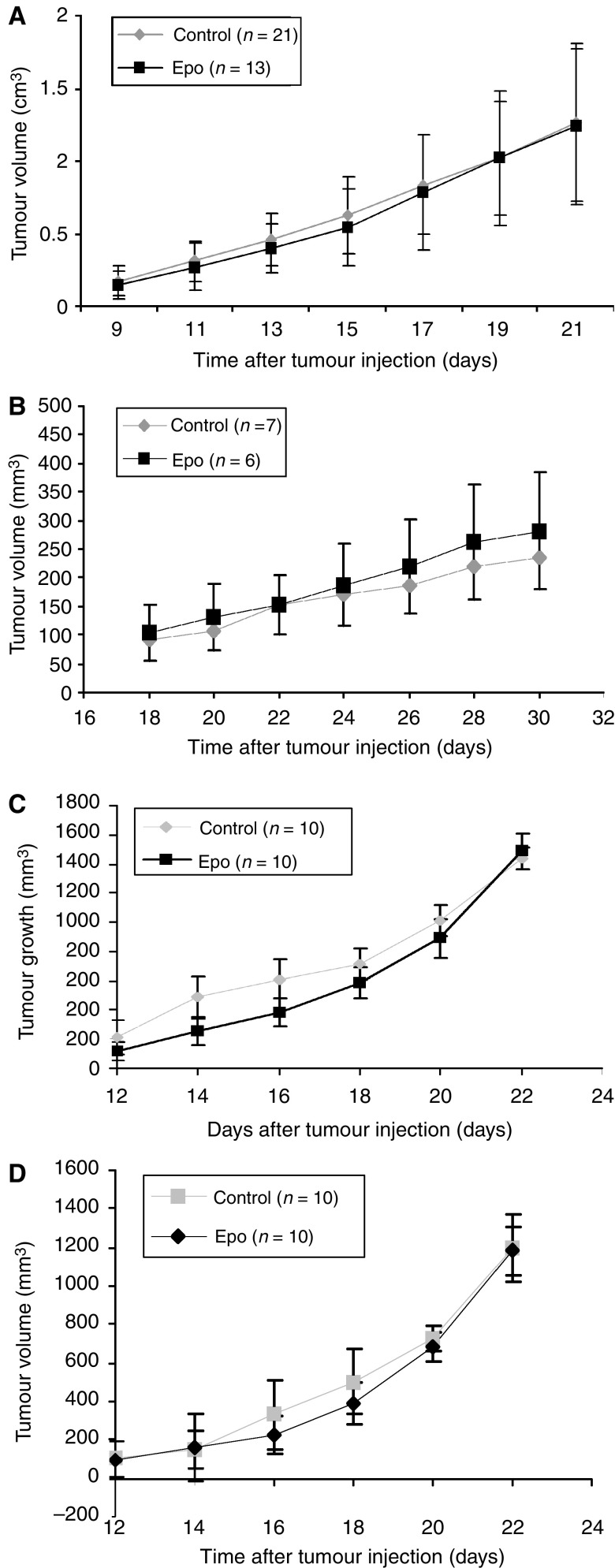
Volume of tumours grown in BALB/C mice and Fischer 344 rats. CT-26 flank tumours (**A**) were grown in the right flank of female BALB/C mice, R3230Ac flank tumours (**B**) were grown in the right flank of female Fischer 344 rats, and FaDu (**C**) and HCT-116 (**D**) flank tumours were grown in the right flank of female athymic mice. rEpo was administered thrice weekly at dose of 2000 units kg^−1^ for a total of six doses of rEpo after tumours reached 100–200 mm^3^. Control-treated and rEpo-treated tumour volumes were similar for all tumour types. Shown are mean values±standard error of the mean (s.e.m.). *T*-test for independent samples with two-tailed *P*-value.

**Figure 3:**
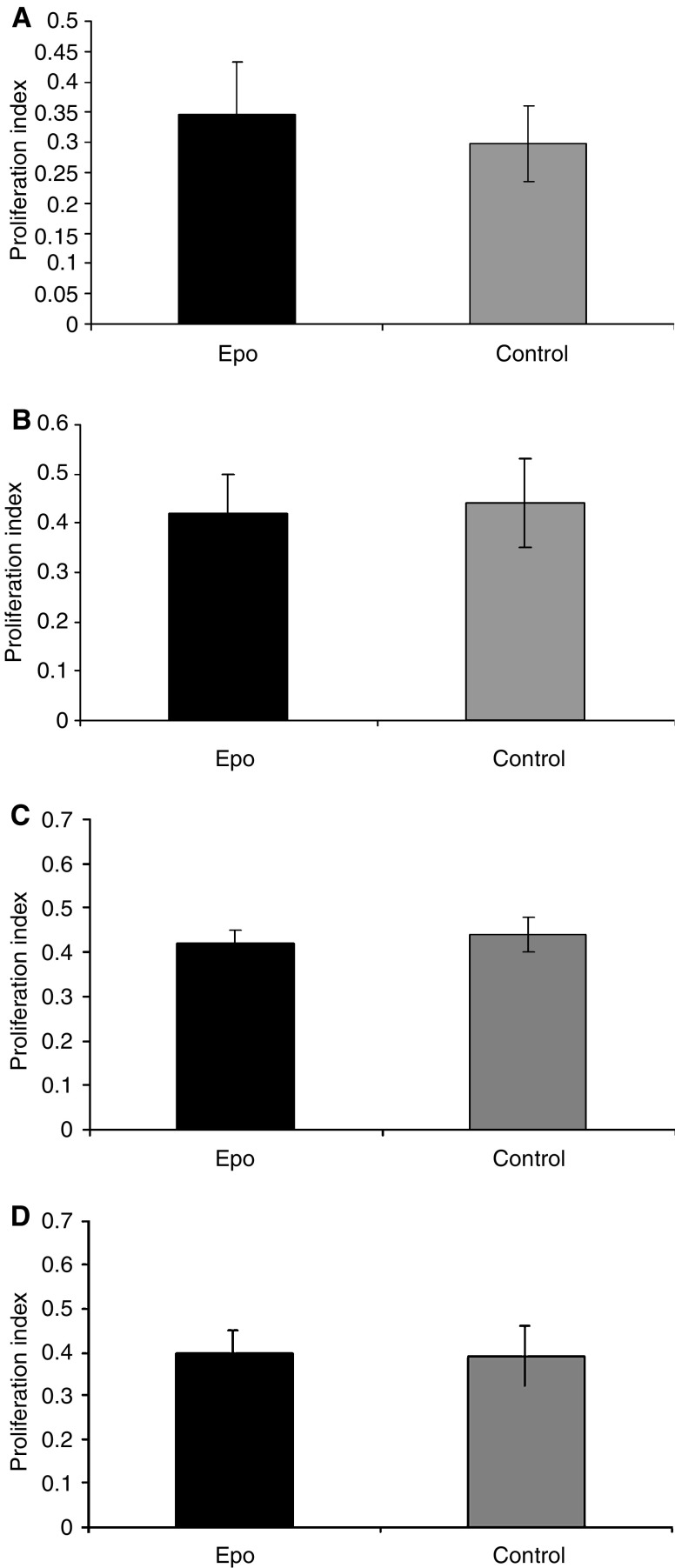
Effect of rEpo on proliferation rate of tumour cells. Sections were taken from (**A**) CT-26, (**B**) R3230, (**C**) FaDu, and (**D**) HCT-116 tumours and stained for Ki-67. There was no statistical difference between the mean proliferation index of the rEpo-treated group and that of the control-treated group in any tumour model (*P*=0.14, 0.23, 0.09, and 0.11, respectively).

Tumour growth was observed in all rats transplanted with R3230 tumour cells and mice transplanted with CT-26, HCT-116, and FaDu tumour cells. Tumour volumes were similar between the EPO-treated and control groups in each of the four tumour cell lines throughout the length of the study (Figure 2A–D). As measured by Ki-67 immunohistochemical staining, there was no statistical difference between the mean proliferation indices between the EPO-treated and control groups (Figure 3) in any of the tumour cell lines.

### Epo does not stimulate tumour angiogenesis

**Figure 4:**
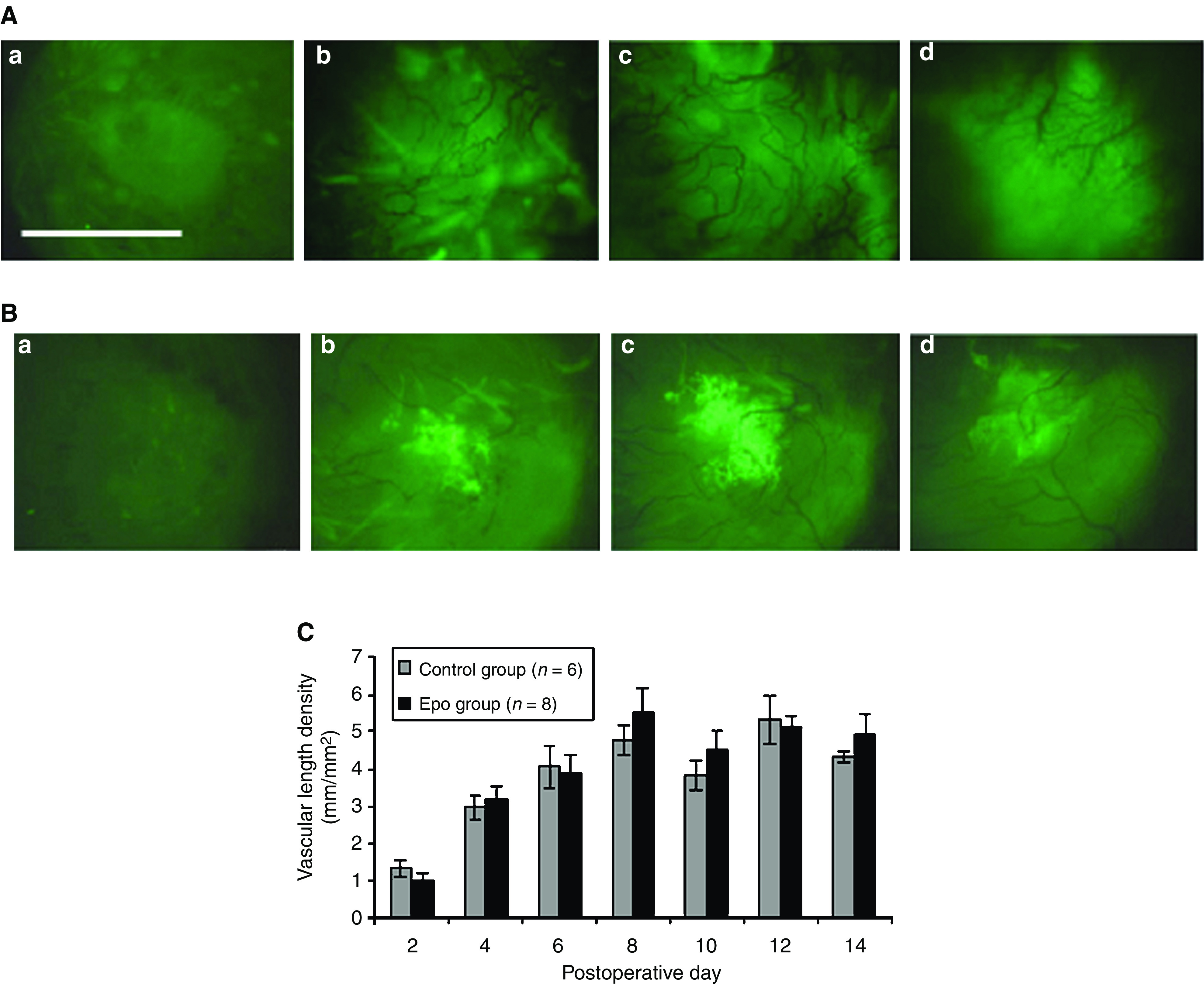
Intravital microscopy of mammary window chamber. Shown are a series of representative images from a rat in the control group (**A**) and the rEpo-treated group (**B**) and the effects of rEpo on tumour vascularity (**C**). Shown images from the mammary window chamber were taken at postoperative day 2 (a), 6 (b), 8 (c), and 14 (d). On postoperative day 2, the tumour size is relatively small, and little angiogenesis has occurred. By postoperative day 6, the tumour has increased in size and amassed a considerable vasculature, which continues to increase until around postoperative day 12–14, when the tumour and associated vasculature begin to reach a plateau. Vascular length density (VLD) was calculated for each animal at each time point. At all time points, there was no statistical difference (*P*>0.05) in the VLD of the treatment and control groups. Values represent the mean of each treatment group±standard error of the mean (s.e.m.) at each time point. Scale bar=300 m.

There was no statistical difference in VLD between the EPO-treated and control groups (Figure 4) at any timepoint (*P*>0.05 at all timepoints). All 20 rats tolerated the surgery well and were healthy immediately postoperatively. At postoperative day 14, 14 of 20 rats retained their mammary windows and had tumours that could be visualized. This represents a success rate of 70%, consistent with that of Shan (Shan *et al*, 2003). Six animals were unevaluable due to: (1) rats that pulled off their windows (*n*=2), (2) lack of tumour growth (*n*=3), or (3) infection/inflammation that prevented tumour visualization (*n*=1).

## DISCUSSION

Recombinant Epo has recently been shown to improve tumour oxygenation; its mechanism has not been determined. Tumour oxygenation is dependent on a delicate balance between oxygen delivery and oxygen consumption. Previous preclinical work in this area, all done *in vitro*, has implicated rEpo as promoting tumour growth (Westenfelder and Baranowski, 2000; Yasuda *et al*, 2002) and/or being proangiogenic (Anagnostou *et al*, 1990; Carlini *et al*, 1995; Ribatti *et al*, 1999; Acs *et al*, 2001; Yasuda *et al*, 2002). However, no studies have looked at the *in vivo* effects of therapeutic levels of rEpo on these two processes.

Several studies have suggested that Epo might stimulate angiogenesis by acting to promote proliferation and/or migration of endothelial cells (Anagnostou *et al*, 1990; Ribatti *et al*, 1999), inhibiting apoptosis of endothelial cells (Carlini *et al*, 1999), or by increasing MMP-2 secretion (Ribatti *et al*, 1999). rEpo has also been shown to increase the number of circulating endothelial cells and endothelial cell progenitors in preclinical models (Monestiroli *et al*, 2001) and human breast cancer and lymphoma models (Mancuso *et al*, 2001). Our studies demonstrate the importance of *in vivo* modeling of rEpo's effects, as we found no effects of rEpo on tumour cell proliferation, tumour growth or angiogenesis.

Previous rEpo study results may be limited in their application by the fact that they examined the effects of rEpo on one particular cell line. In addition, previous studies used supratherapeutic concentrations of recombinant Epo (5–100 U ml^−1^; Carlini *et al*, 1995), as compared to levels of Epo achieved clinically to correct hematocrit (1.7–3.6 U ml^−1^; (Carlini *et al*, 1995)). Also, the expression of Epo receptor on the tumour or endothelial cell line that was used was not determined and/or the rEpo was administered over a short period of time.

Our study is strengthened by the fact that we used four separate tumour lines that express the Epo receptor and treated the animals over an extended period. While the aim of this study was to determine the effects of exogenous rEpo on two biologic processes that directly affect tumour oxygenation (tumour growth and angiogenesis), intra-tumoural production of Epo might also influence tumour behavior in an autocrine/paracrine fashion. Evidence for a functional Epo-EpoR system has been reported by several groups: intratumoural and intraperitoneal injections of solube EpoR or neutralizing monoclonal antibody to EpoR resulted in tumour growth delay and inhibition of angiogenesis (Yasuda *et al*, 2001, 2003; Arcasoy *et al*, 2002).

There have been no reports to date precisely analyzing the angiogenic potential of therapeutic rEpo in an *in vivo* tumour model. Our laboratory has extensively utilized the rodent dorsal skin flap window chamber model in determining real-time, sequential angiogenic effects of various agents (Li *et al*, 2000). A limitation of the dorsal skin fold model, however, has been the necessity of growing tumours in an ectopic site. Recently, our laboratory has developed an orthotopic breast window, as described by Shan (Shan *et al*, 2003). It has been established that tumour microenvironment plays an influential role in the behavior of the tumour itself and that tumours originating from different tissues differ in their phenotypes, biological behaviors, and responses to therapeutic agents (Fidler *et al*, 1994). When tested in this highly translational *in vivo* model, rEpo did not demonstrate any proangiogenic activity.

Finally, our studies offer insight into possible clinical effects of rEpo on patients receiving the drug to treat anemia. Two recent clinical trials (Henke *et al*, 2003; Leyland-Jones, 2003) have suggested that rEpo might negatively impact treatment outcome in cancer patients receiving chemotherapy. Additionally, *in vitro* evidence suggests that rEpo may induce a cytoprotective phenotype in certain tumour types, which could translate into resistance to certain chemotherapeutics (Belenkov *et al*, 2004; McBroom *et al*, 2005). However, a recent meta-analysis of rEpo used in both patients with anemia and without anemia suggests a strong trend towards a survival advantages in favor of the use of rEpo (Bohlius *et al*, 2005). Although not combined with chemotherapy or radiation therapy, the present study is the first to provide preclinical *in vivo* insight into this seemingly confusing clinical situation. In addition, our study suggests that recent clinical results using rEpo in nonanemic patients may not be due to the direct effects of the drug on the tumour, as we did not detect any tumour growth enhancement with rEpo. Our findings warrant further preclinical and clinical investigation of rEpo in order to further clarify the risks of its use as well as optimize its known benefits.
